# QTL-Seq identified a genomic region on chromosome 1 for soil-salinity tolerance in F_2_ progeny of Thai salt-tolerant rice donor line “Jao Khao”

**DOI:** 10.3389/fpls.2024.1424689

**Published:** 2024-08-26

**Authors:** Prasit Khunsanit, Navarit Jitsamai, Nattana Thongsima, Supachitra Chadchawan, Monnat Pongpanich, Isabelle M. Henry, Luca Comai, Duangjai Suriya-Arunroj, Itsarapong Budjun, Teerapong Buaboocha

**Affiliations:** ^1^ Program in Biotechnology, Faculty of Science, Chulalongkorn University, Bangkok, Thailand; ^2^ Center of Excellence in Molecular Crop, Department of Biochemistry, Faculty of Science, Chulalongkorn University, Bangkok, Thailand; ^3^ Program in Bioinformatics and Computational Biology, Graduate School, Chulalongkorn University, Bangkok, Thailand; ^4^ Center of Excellence in Environment and Plant Physiology, Department of Botany, Faculty of Science, Chulalongkorn University, Bangkok, Thailand; ^5^ Omics Sciences and Bioinformatics Center, Faculty of Science, Chulalongkorn University, Bangkok, Thailand; ^6^ Department of Mathematics and Computer Science, Faculty of Science, Chulalongkorn University, Bangkok, Thailand; ^7^ Department of Plant Biology and Genome Center, University of California, Davis, Davis, CA, United States; ^8^ Rice Department, Ministry of Agriculture and Cooperation, Bangkok, Thailand

**Keywords:** quantitative trait loci, bulk segregant analysis, QTL-seq, QTL mapping, QTL validation, marker-assisted selection, rice, salt tolerance

## Abstract

**Introduction:**

Owing to advances in high-throughput genome sequencing, QTL-Seq mapping of salt tolerance traits is a major platform for identifying soil-salinity tolerance QTLs to accelerate marker-assisted selection for salt-tolerant rice varieties. We performed QTL-BSA-Seq in the seedling stage of rice from a genetic cross of the extreme salt-sensitive variety, IR29, and “Jao Khao” (JK), a Thai salt-tolerant variety.

**Methods:**

A total of 462 F_2_ progeny grown in soil and treated with 160 mM NaCl were used as the QTL mapping population. Two high- and low-bulk sets, based on cell membrane stability (CMS) and tiller number at the recovery stage (TN), were equally sampled. The genomes of each pool were sequenced, and statistical significance of QTL was calculated using QTLseq and G prime (G′) analysis, which is based on calculating the allele frequency differences or Δ(SNP index).

**Results:**

Both methods detected the overlapping interval region, wherein CMS-bulk was mapped at two loci in the 38.41–38.85 Mb region with 336 SNPs on chromosome 1 (*qCMS1*) and the 26.13–26.80 Mb region with 1,011 SNPs on chromosome 3 (*qCMS3*); the Δ(SNP index) peaks were −0.2709 and 0.3127, respectively. TN-bulk was mapped at only one locus in the overlapping 38.26–38.95 Mb region on chromosome 1 with 575 SNPs (*qTN1*) and a Δ(SNP index) peak of −0.3544. These identified QTLs in two different genetic backgrounds of segregating populations derived from JK were validated. The results confirmed the colocalization of the *qCMS1* and *qTN1* traits on chromosome 1. Based on the CMS trait, *qCMS1/qTN1* stably expressed 6%–18% of the phenotypic variance in the two validation populations, while *qCMS1/qTN1* accounted for 16%–20% of the phenotypic variance in one validation population based on the TN trait.

**Conclusion:**

The findings confirm that the CMS and TN traits are tightly linked to the long arm of chromosome 1 rather than to chromosome 3. The validated *qCMS-TN1* QTL can be used for gene/QTL pyramiding in marker-assisted selection to expedite breeding for salt resistance in rice at the seedling stage.

## 1 Introduction

Soil salinity poses a significant threat to food security and human nutrition, as it can be caused by both natural soil salinization occurrences and artificial conditions (Amoah et al., 2020; Kumar and Sharma, 2020; Lei et al., 2020). In the northeast part of Thailand, which is the primary region for rice cultivation, rice yields are relatively low. One of the key factors contributing to this decline in rice yield is soil salinity originating from underground rock salt and seemingly influenced by rainfall patterns (Yang et al., 2022). Salt-prone regions in the northeast cover 1.84 million hectares, representing 16% of the rainfed lowland area of this part of Thailand (Punyawaew et al., 2016; Vanavichit et al., 2018). Soil affected with 50 mM NaCl can result in rice (*Oryza sativa*) yield reduction by up to 50% (Wu et al., 2020) and poses a significant limitation to all stages of rice cultivation by limiting soil water absorption (osmotic stress), increasing the Na^+^/K^+^ ratio within the cell (ionic toxicity) through ion uptake by membrane transporters, and initiating oxidative stress (Bonilla, 2002; Munns and Tester, 2008). Rice has been regarded as a preeminent model for exploring molecular genetics in cereal crops (Khush and Brar, 2001; Haque et al., 2021). The utilization of high-throughput sequencing to sequence the rice genome has resulted in the identification of a considerable number of mutations present within the genome (Lekklar et al., 2019b). These mutations are indicative of high genetic diversity within the rice genome. Single-nucleotide polymorphisms (SNPs) and insertion/deletion (InDel) events occur with a high frequency across both coding and non-coding regions (Jain et al., 2014). These polymorphisms are highly diverse and can be utilized as genetic markers (Ganal et al., 2009; Rajcan et al., 2011; Gimhani et al., 2016). Consequently, a novel line of rice cultivars with tolerance to salinity has been developed by utilizing a range of genetic resources aimed at mitigating susceptibility to salinity (Quan et al., 2018).

The regulation of plant phenotype is predominantly mediated by polygenic factors and their interaction with the external environment. The salt tolerance trait is influenced by both major and minor quantitative trait loci (QTLs) (Mackill and Ni, 2001), with each having a small effect (Takagi et al., 2013). The identification of QTLs is still challenging when investigating the multiple genes that affect salt tolerance in rice. QTL mapping is a forward genetics method used to identify the location of QTLs in the genome, which can be employed alongside bulk segregant analysis (BSA) and genome sequencing (Majeed et al., 2022). QTL-BSA mapping is a rapid and cost-effective technique (Zegeye et al., 2018; Dai et al., 2022; de la Fuente Cantó and Vigouroux, 2022) for detecting novel QTLs for salinity tolerance by establishing a biparental population and mapping it to the F_2_ segregating population (Sun et al., 2019; Pundir et al., 2021). SNPs are widespread in the genome due to its many polymorphisms and are effectively present in genes/QTLs that can be frequently linked to important traits. Compared with conventional QTL mapping, QTL-Seq offers the possibility to detect the abundance of DNA polymorphisms, such as SNPs and InDels across the genome, which are invaluable for use as molecular markers in marker-assisted selection (MAS) (Dwiningsih et al., 2020).

In MAS development, QTLs are mapped and validated (Collard and Mackill, 2008; Acquaah, 2012; Geng et al., 2023). Thus, the first step of MAS development is QTL mapping, which involves the construction of mapping populations and linkage maps. The next step is validation, which is a verification step to confirm the stability of the identified QTLs and includes QTL validation using the population derived from the QTL mapping. The marker validation process is performed using important breeding material before marker-assisted breeding. In rice, both wild and cultivated rice, QTL mapping has been performed to identify novel QTLs for salt-stress tolerance (Bizimana et al., 2017; Rahman et al., 2017; Quan et al., 2018; Kong et al., 2021; Singh et al., 2021; Xie et al., 2022; Yuan et al., 2022). Salt tolerance is a complex trait controlled by multiple genes. Identifying and characterizing the causative genes to the end of clarifying their molecular mechanisms is crucial for improving new cultivars (Flowers, 2004; Reddy et al., 2017). Additionally, to develop new cultivars with enhanced yield and tolerance to abiotic stress, genetic variation at all levels, both within and between species, has been employed in breeding schemes; however, the limited availability of germplasm for salt tolerance that can be crossed without associated yield penalty has led to slow progress in developing salt-tolerant cultivars (Ashraf and Munns, 2022). For Thai rice, only a few local germplasms have been used in rice breeding for salt tolerance traits. Our previous study revealed that “Jao Khao” (JK), a Thai domestic germplasm rice variety, has the capacity for high yields under salinity stress (Lekklar et al., 2019a; Habila et al., 2022). Therefore, JK is a promising candidate for facilitating the transmission of salt tolerance traits; hence, we use it as a donor line for salt tolerance traits.

In this study, we aimed to develop molecular markers for soil-salinity tolerance by identifying QTLs in the F_2_ progeny derived from the donor rice line, JK, using QTL-Seq mapping combined with BSA. The identified QTLs were then validated in validation populations obtained from two different genetic backgrounds.

## 2 Materials and methods

### 2.1 Construction of the QTL mapping population

**Figure 1:**
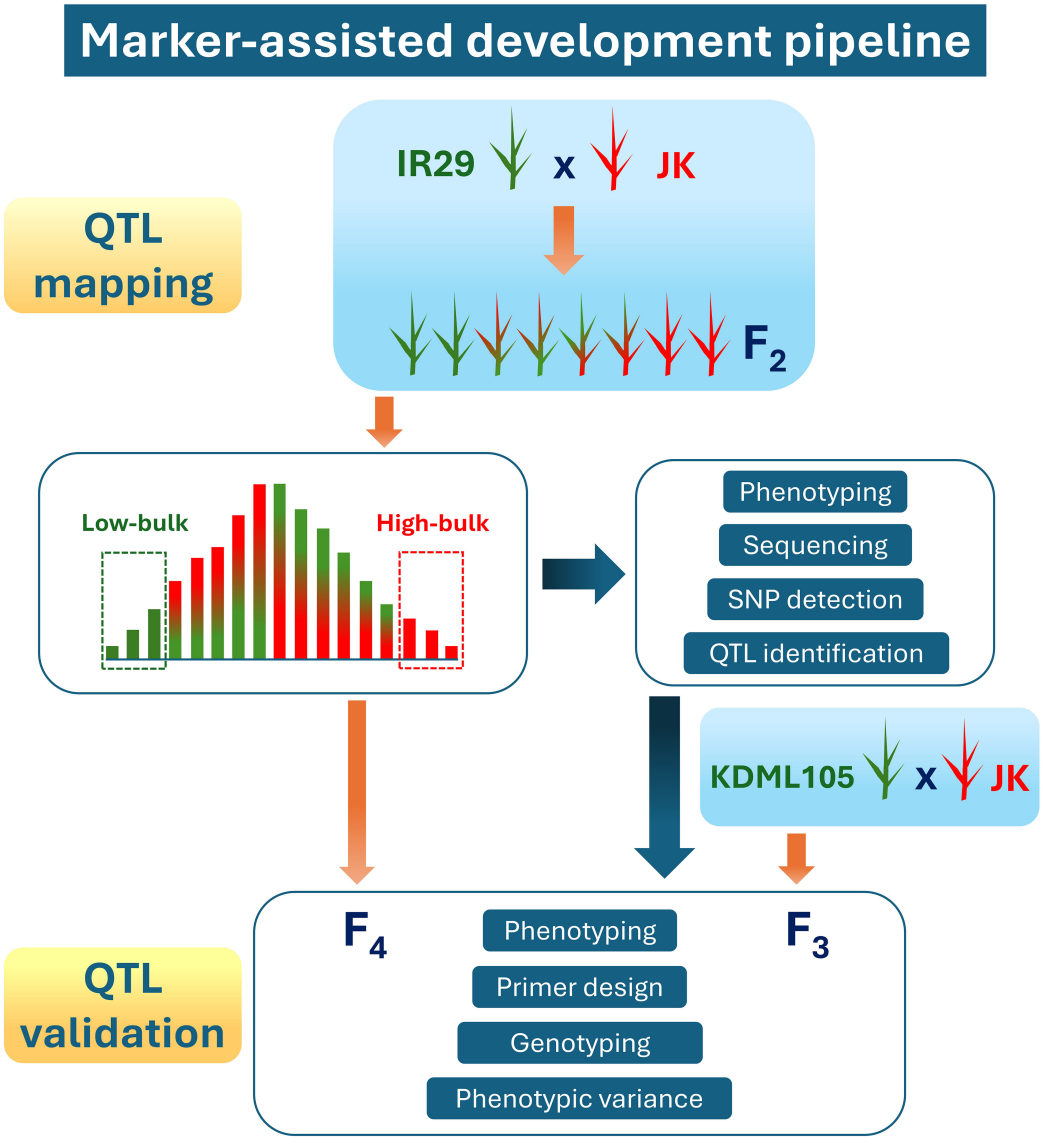
Marker-assisted development pipeline used in this study.

According to our previous study on 206 varieties of Thai rice under salt stress in the heading stage (Lekklar et al., 2019a), the JK variety showed high tillers per plant and filled grain per plant stability indexes compared with those of “Pokkali” (a standard salt-tolerant cultivar) (Habila et al., 2022). Consequently, JK was used as a donor line in the cross with the standard salt-sensitive cultivar IR29. The marker-assisted development pipeline used in the current study, including QTL mapping and validation, is illustrated in **Figure 1**. The F_1_ progeny were produced and then self-pollinated to generate more than 10,000 seeds of the F_2_ population. These experiments were conducted at the Nakhon Ratchasima Rice Research Center, Rice Department, Ministry of Agriculture and Cooperatives, Thailand.

### 2.2 Phenotyping under salt conditions and selecting bulked extreme pools

Rice seeds were randomly selected from the F_2_ population, soaked in water for germination, and transplanted into soil in a 4-inch pot (one seedling per pot). Next, a total of 462 3-week-old rice seedlings were subjected to 115 mM NaCl treatment for 3 days (13.1 dS m^−1^). After this treatment period, the concentration of the saline solution was increased to 160 mM NaCl for 7 days (18.6 dS m^−1^). Then, on day 7, cell membrane stability (CMS) was determined, and tap water was used thereafter to remove salt to re-establish normal conditions. After 10 days of recovery, the tiller number per plant (TN) was counted. CMS was determined based on electrolyte leakage, which was determined via electrical conductivity (EC) measurement. Approximately 1.5 mg of fully expanded leaf tissues were selected, weighed, cut into small 1- to 2-cm pieces, soaked in 10 mL of deionized water, and incubated at 25°C for 4–5 h. Thereafter, the initial electrical conductivity (EC1) was determined. To disturb the whole cell, the samples were autoclaved at 121°C for 20 min and then cooled, after which the final electrical conductivity (EC2) was measured. CMS was calculated as CMS = 100 × [1 − (EC1/EC2)] (Habila et al., 2021). Two sets of phenotypes were separately arranged to select the pool of opposite phenotypes: CMS-bulk contained 21 plants each of high- or low-CMS, and TN-bulk contained 28 plants each of high- or low-TN.

### 2.3 Genomic DNA extraction, library preparation, and sequencing

Fresh leaf samples were collected before the rice seedlings were subjected to salt-stress conditions. Genomic DNA (gDNA) was extracted from the fresh leaves using a gDNA extraction kit (Geneaid Biotech Ltd., New Taipei City, Taiwan). Individual plants from all four groups of the two phenotypes (high- and low-CMS and high- and low-TN) were used to construct gDNA libraries, which were pooled in equal amounts for each group. To prepare the whole-genome library, genomic DNA was fragmented with dsDNA Fragmentase (New England Biolabs, Ipswich, MA). The resulting sheared DNA was modified with an End Repaired enzyme (New England Biolabs), and deoxyadenosine was added to the 3′ end using a Klenow fragment (New England Biolabs). Each library had unique DNA barcodes (Bioo Scientific, Austin, TX) joined to the DNA using DNA ligase (New England Biolabs). The short sequence reads obtained from the Illumina Sequencer (Illumina, San Diego, CA, USA) were demultiplexed and trimmed using Trimmomatic-0.39 (available at http://www.usadellab.org/cms/index.php?page=trimmomatic) (Bolger et al., 2014). The raw reads from each extreme pool were aligned against the reference genome by the Burrow-Wheeler Aligner (version 0.5.7–1) (Li and Durbin, 2010) using the rice reference genome version IRGSP-1.0 downloaded from the EnsemblPlants database (https://plants.ensembl.org/index.html) (Kawahara et al., 2013; Matsumoto et al., 2016) and indexed by SAMtools (Li et al., 2009). The mapped reads were processed using Picard (https://broadinstitute.github.io/picard/). Variants were called and filtered using a genome analysis toolkit (GATK; version 3.3-0) (McKenna et al., 2010).

### 2.4 QTL mapping using QTLseqr

The QTLs were mapped using both the QTL-seq method and the G prime method in the R package “QTLseqr” (Magwene et al., 2011; Mansfeld and Grumet, 2018). The identified SNPs were filtered using the following criteria: reference allele frequency = 0.4, minimal total depth = 100, maximum total depth = 400, depth difference between bulks = 60, minimum sample depth = 40, and minimum genotype quality = 99. The SNP index at any allele location was compared with that of reference alleles (Nipponbare). The SNP index was calculated as SNP index_i_ = the alternate allele depth/total read depth, where *i* is the location of the SNP. In a given genomic interval, the distribution of Δ(SNP index) between the bulks was estimated using a 50-kb (for QTL-seq) or 1-Mb (for G prime) sliding window. The regions where the Δ(SNP index) passed the confidence intervals of 95% and 99% according to the statistical simulations were referred to as regions harboring the major QTLs responsible for the differences between the bulks (Takagi et al., 2013; Itoh et al., 2019).

### 2.5 Establishment of the validation population and phenotyping under soil salinity stress

Two progeny validation populations were used to assess the consistency of the identified QTLs. To this end, the single-seed descent (SSD) method was established. First, the F_4_ population was produced from the F_2_ progeny derived from crossing JK with IR29 from the prior QTL mapping population. Second, the population for marker validation was established using F_3_ from a cross between JK and “Khao Dawk Ma Li 105” (KDML105), a relatively salt-sensitive Thai rice cultivar. These experiments were performed at the Nakhon Ratchasima Rice Research Center. To establish the validation population, seeds from the F_4_ and F_3_ populations were sown to produce approximately 800 and 1,200 seedlings, respectively. The stress and recovery conditions were given as previously described. The phenotypes were assessed in two stages: during stress, measuring CMS and tiller number_stress (TN_s), and during recovery, measuring tiller number_recovery (TN_r). CMS and TN were determined as previously described.

### 2.6 Primer design for the identified QTL regions

PCR using allele-specific primers (ASPs) was performed based on the significant SNPs identified in the QTLs. These primers were designed to bind the regions containing SNPs that can clearly distinguish between the salt-tolerant allele (JK) and the salt-sensitive alleles (IR29 and KDML105). We used at least 250 bp covering the target SNPs to design the ASP with approximately 30-bp gap between each product by the WebSNAPER software (https://pga.mgh.harvard.edu/cgi-bin/snap3/websnaper3.cgi). A mismatch base was added within the three nucleotides adjacent to the 3′ terminus to improve the efficacy of the differential PCR. Reportedly, a single base mismatch (SNP) can cause false-positive detection; therefore, increasing the number of artificial mismatch bases can reduce false-positive results (Liu et al., 2012; Kim et al., 2016).

### 2.7 Genomic DNA extraction, PCR amplification, and genotyping

gDNA was isolated from young leaf tissue according to the CTAB procedure (Aboul-Maaty and Oraby, 2019). The DNA concentration was diluted to 100 ng/mL, and the quality was determined using a UV spectrophotometer (Eppendorf, Hamburg, Germany). To identify the unique molecular marker, an ASP was tested on the F_3_ or F_4_ progenies. The PCR mixture (10 µL) contained 1 µL of gDNA, 0.5 µL of each primer (0.5 µM), 5 µL of 2×Tag Mastermix PCR buffer (Vivantis Technologies, Shah Alam, Malaysia), and 3 µL of ddH_2_O. First, a step gradient PCR program was used to determine the proper annealing temperature of each primer. The program was set to comprise an initial denaturation at 95°C for 5 min, followed by 35 cycles of amplification consisting of denaturation at 95°C for 25 s, annealing at 49°C for 30 s, and extension at 72°C for 35 s. The final extension was conducted at 72°C for 5 min, followed by a cooling step at 20°C for 5 min. The PCR amplicon was combined with a 6× tracking dye and subjected to electrophoresis using 2% agarose gel and run in 1× TBE buffer at 100 mV for 30 min. The gels were recorded using a molecular imaging system (Amersham ImageQuant 800, GE Healthcare, UK) to assess and score the bands.

### 2.8 Phenotypic variance analysis

QGene software (version 4.4.0) (Joehanes and Nelson, 2008) was used to perform single marker analysis of phenotype and genotype correlations. The correlations were expressed as *R* ^2^ (%) and used to explain the proportion of the phenotypic variation explained (PVE) by the QTLs of the progenies. The mean phenotypic value for each line was determined using individuals with similar genotypes, with a minimum of three replicates. Furthermore, descriptive and Pearson analyses were performed using SPSS (SPSS Inc., Chicago, IL, USA) (George and Mallery, 2019). R (R Foundation for Statistical Computing, Vienna, Austria) was used to construct graphs (Team, 2020).

## 3 Results

### 3.1 Phenotype of the F_2_ mapping population

**Figure 2:**
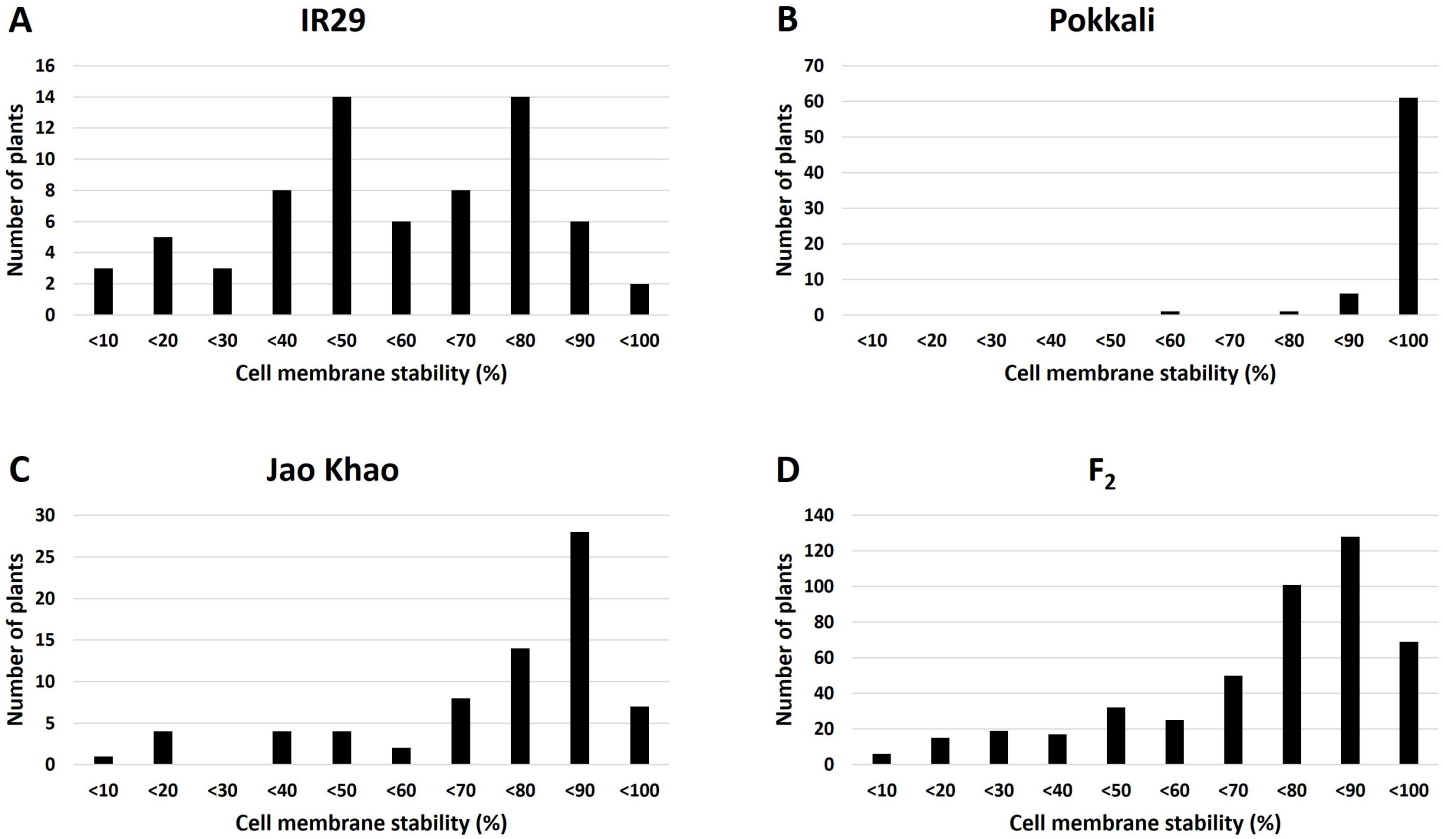
Distribution of cell membrane stability under salt-stress treatment in **(A)** IR29 (*n* = 69), **(B)** “Pokkali” (*n* = 69), **(C)** “Jao Khao” (*n* = 72), and **(D)** the F_2_ population (*n* = 462).

**Figure 3:**
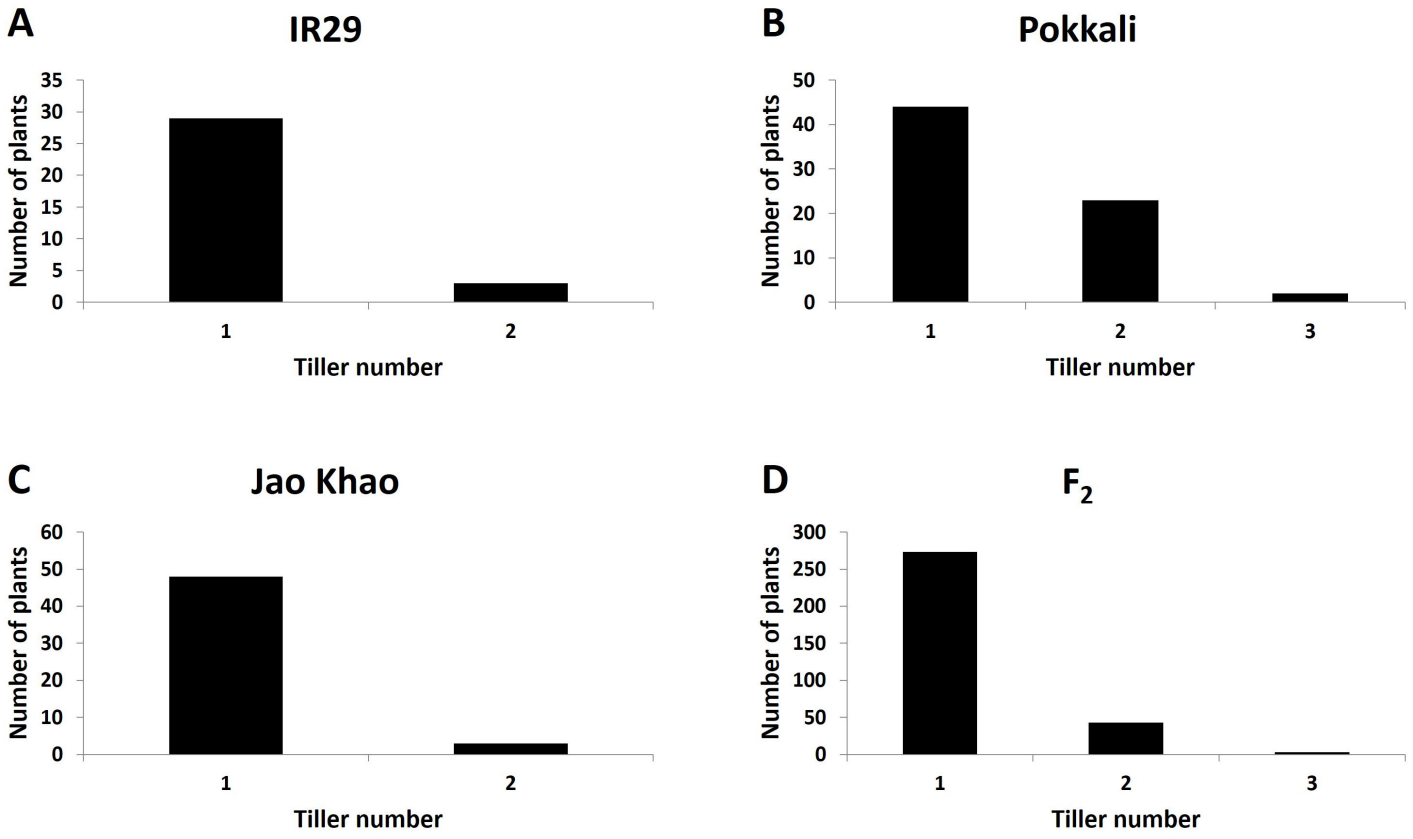
Distribution of tiller number under salt-stress treatment in **(A)** IR29 (*n* = 69), **(B)** “Pokkali” (*n* = 69), **(C)** “Jao Khao” (*n* = 72), and **(D)** the F_2_ population (*n* = 462).

Phenotyping of the F_2_ population derived from a cross between the extreme salt-sensitive variety IR29 and JK, a Thai salt-tolerant variety, was performed in two phases: CMS and TN_s traits were measured in the stressed phase, and the TN_r trait was measured in the recovery phase. The distribution of the CMS values is shown in **Figure 2**. IR29, a standard salt-sensitive line, displayed a bimodal distribution, which exhibited two distinct peaks at 50%–60% and 70%–80% CMS (**Figure 2A**), whereas “Pokkali” a standard salt-tolerant line, mostly exhibited a high CMS (**Figure 2B**). The CMS distribution of F_2_ (**Figure 2D**) was more similar to that of JK, the salt donor line used in the present study (**Figure 2C**). TN demonstrated that almost all varieties had one tiller per plant at the recovery stage after salt treatment. “Pokkali” had a greater proportion of plants with two tillers, and “Pokkali” and F_2_ offspring had a few plants with three tillers per plant (**Figure 3**). The CMS distribution of F_2_ was left-skewed under a normal distribution, and the means of high and low CMS-bulk were 92.84% and 7.05%, respectively (**Supplementary Table 1** and **Supplementary Figures 1A, C**). However, the TN distribution showed the opposite trend, i.e., a right-skewed pattern (**Supplementary Figures 1B, D**), with the means of the high TN-bulk and low TN-bulk being 2 and 0 tillers per plant, respectively.

### 3.2 QTL mapping of the bulked extreme pool combining G′ and QTL-Seq analyses

**Figure 4:**
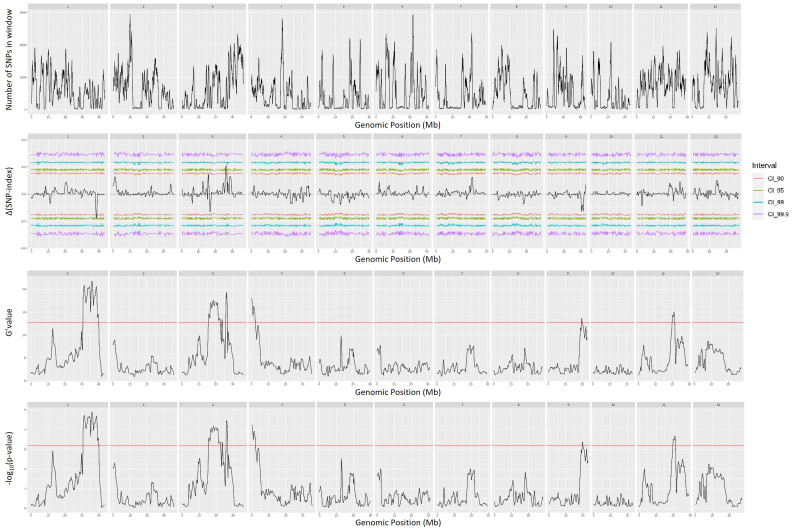
Quantitative trait loci mapping for salt tolerance identified by the cell membrane stability (CMS) of 12 chromosomes, including the number of single-nucleotide polymorphisms (SNPs) in the sliding window, Δ(SNP index), G′ value, and −log_10_(*p*-value).

**Figure 5:**
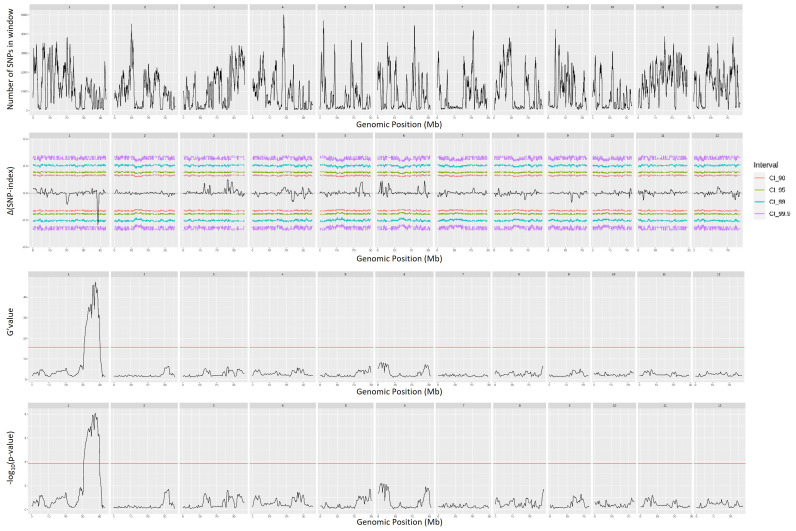
Quantitative trait loci mapping for salt tolerance identified by the tiller number (TN) of 12 chromosomes, including the number of single-nucleotide polymorphisms (SNPs) in the sliding window, Δ(SNP index), G′ value, and −log_10_(*p*-value).

QTL mapping by QTLseqr revealed that for the CMS trait, QTLs were identified on chromosomes 1, 3, 4, 9, and 11 using the G′ method, and to chromosomes 1 and 3 using the QTL-seq method. The overlapping regions between the two methods were between 38.41 and 38.85 Mb on chromosome 1, designated as *qCMS1* (QTL1), and between 26.13 and 26.80 Mb on chromosome 3, designated *qCMS3* (QTL2). These regions contained 336 and 1,011 SNPs, respectively (**Table 1** and **Figure 4**). In both methods, the TN trait was found to be located at an interval of 38.26–38.95 Mb on chromosome 1, designated *qTN1*, and contained 575 SNPs (**Table 2** and **Figure 5**), which overlapped with QTL1.

**Table 1:** Single-nucleotide polymorphism (SNP) peaks associated with cell membrane stability detected by the G′ and QTL-Seq methods.

Method	CHROM	qtl	Start	End	Length	No. of SNPs	Mean no. of SNP/Mb	Peak Δ(SNP-index)	Mean Δ(SNP-index)	Max G′	Mean G′	G′ Std. dev.	Mean *p*-value	Mean *q*-value
G′	1	1	30,617,960	39,961,306	9,343,346	8,393	898	0.058697	0.005874	21.76383	18.97439	2.063647	6.60E-05	0.003042
	3	2	15,570,142	22,687,754	7,117,612	11,812	1,660	0.218607	0.024262	17.62806	15.89095	1.569176	0.000194	0.004124
	3	3	23,582,145	23,878,378	296,233	252	851	0.009252	0.008254	13.49003	13.14229	0.1408	0.00052	0.008267
	3	4	25,872,766	26,814,964	942,198	1,035	1,098	0.313047	0.304769	19.34697	18.5116	0.84721	5.30E-05	0.002777
	4	5	5,605	2,144,214	2,138,609	2,956	1,382	0.09567	0.028044	18.02003	16.09915	1.253965	0.000154	0.003774
	9	6	19,676,861	19,990,807	313,946	170	541	−0.01015	−0.00407	13.64901	13.24721	0.127193	0.000493	0.007963
	11	7	19,861,809	21,359,446	1,497,637	2,943	1,965	0.102444	0.072623	15.00849	14.41921	0.358964	0.000285	0.005587
QTL-seq	1	1	38,413,342	38,853,277	439,935	336	764	−0.270955	−0.260786					
	3	2	26,139,128	26,800,598	661,470	1,011	1,528	0.312797	0.306599					

**Table 2:** Single-nucleotide polymorphism (SNP) peaks detected by the G′ and QTL-Seq methods based on tiller number at the recovery stage.

Method	CHROM	qtl	Start	End	Length	No. of SNPs	Mean no. of SNP/Mb	Peak Δ(SNP-index)	Mean Δ(SNP-index)	Max G′	Mean G′	G′ Std. dev.	Mean *p*-value	Mean *q*-value
G′	1	1	30,724,565	40,119,862	9,395,297	15,326	1,631	−0.35449	−0.00141	47.38279	33.21672	8.066812	3.64E-06	0.000208
QTL-seq	1	1	38,261,181	38,955,356	694,175	575	828	−0.35449	−0.33043					

### 3.3 Phenotypic evaluation of the two populations for QTL validation

**Figure 6:**
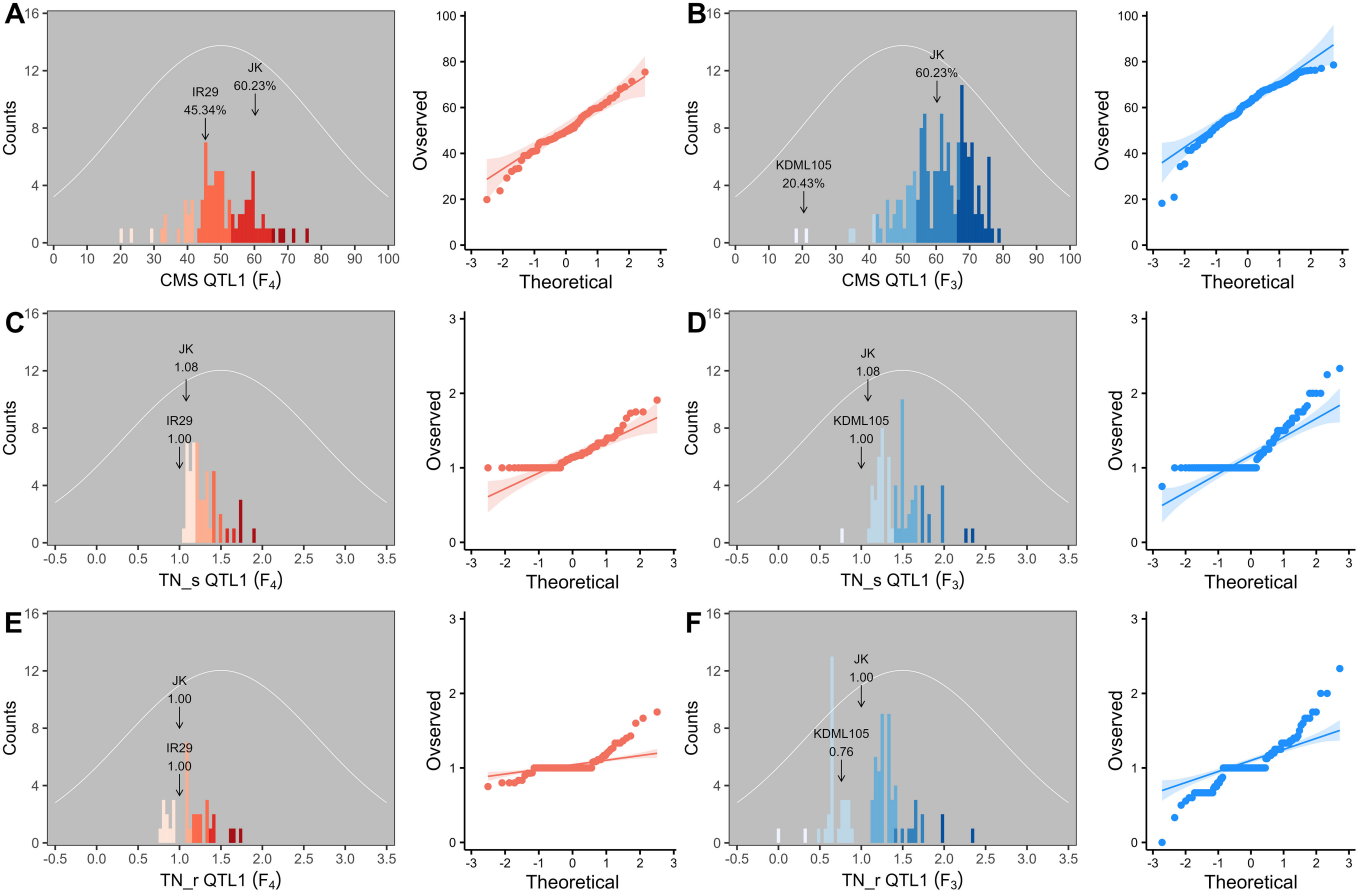
Frequency distribution and quantile–quantile (Q–Q) plots of QTL1 in two validation populations. Cell membrane stability (CMS) **(A, B)**, tiller number (TN)_s **(C, D)**, and TN_r **(E, F)**. F_4_ progeny, *n* = 82; F_3_ progeny, *n* = 153. The color gradient was cut at five levels for each parameter, and the number of bins was set to 100. The arrow indicates the means of the parent cultivars, and the normal distribution curve is shown as a white line.

**Figure 7:**
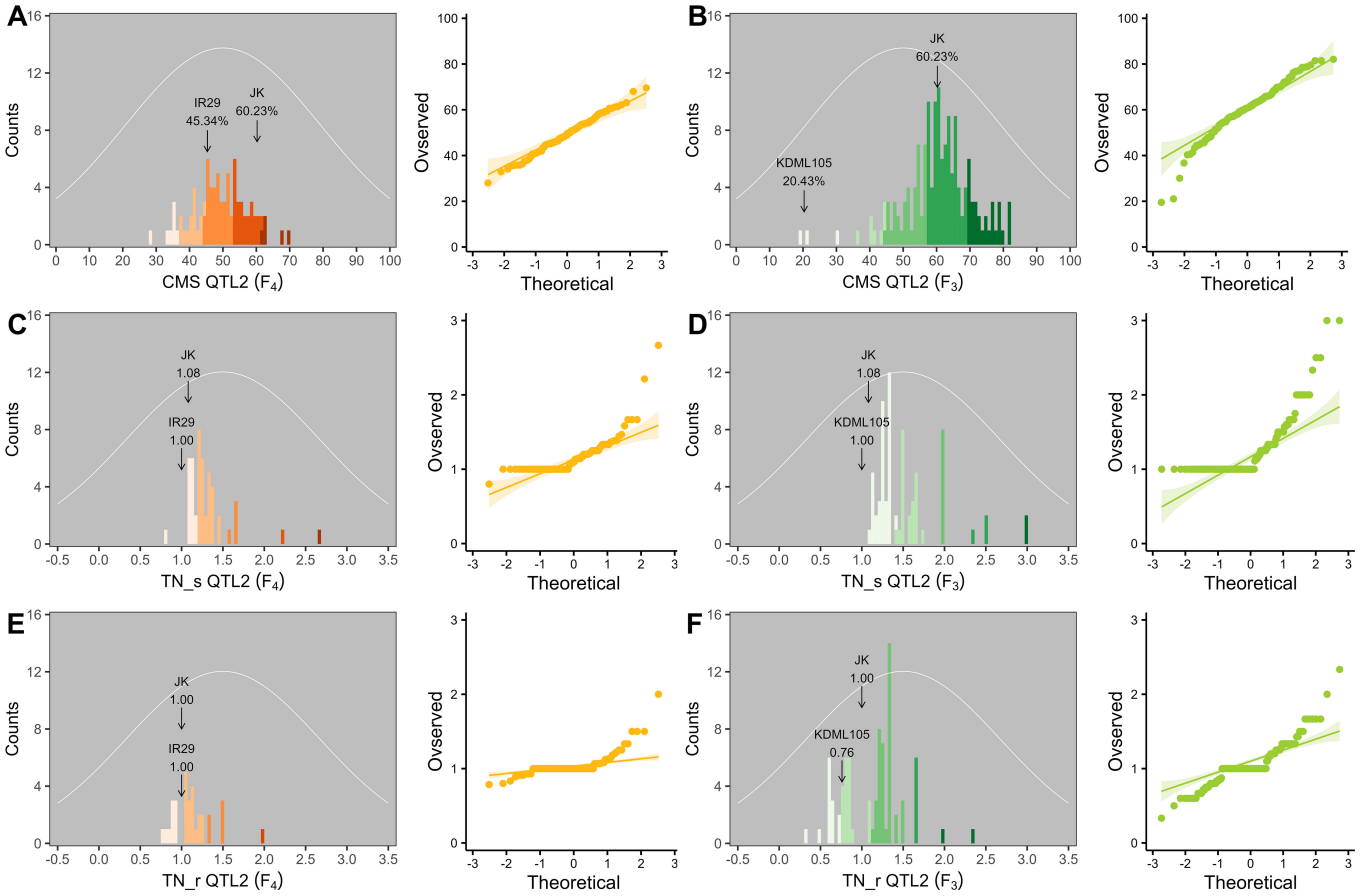
Frequency distribution and quantile–quantile (Q–Q) plots of QTL2 in two validation populations. Cell membrane stability (CMS) **(A, B)**, tiller number (TN)_s **(C, D)**, and TN_r **(E, F)**. F_4_ progeny, *n* = 84; F_3_ progeny, *n* = 159. The color gradient was cut at five levels for each parameter, and the number of bins was set to 100. The arrow indicates the means of the parent cultivars, and the normal distribution curve is shown as a white line.

Two validation populations, F_4_ [derived from F_2_ (JK × IR29) of the previously mapped population] and F_3_ [F_3_ (JK × KDML105) constructed later] progenies, were used to validate the identified QTLs, i.e., QTL1 and QTL2 on chromosomes 1 and 3, respectively. The validation populations were established using different genetic backgrounds based on the JK cultivar. JK is a domestic Thai germplasm rice that shows high-yield performance at the heading stage (Lekklar et al., 2019a), and its TN increases by more than 50% after salt stress at the vegetative stage (Habila et al., 2021). On the other hand, KDML105 is a commercial Thai rice with a jasmine-like aroma that has a high cooking/eating quality profile (Vanavichit et al., 2018; Pamuta et al., 2022). For QTL validation, we considered three phenotype parameters, namely, CMS, TN_s, and TN_r. The phenotypic values for the F_4_ and F_3_ progenies and validation population parents are presented in the **Supplementary Materials** (**Supplementary Tables 2**–**6**, respectively). The summaries of the phenotypic values of the three traits considering QTL1 and QTL2 are shown in **Table 3** and **Figure 6**, and **Table 4** and **Figure 7**, respectively. Most traits were approximately normally distributed. The average CMS values of parent JK, IR29, and KDML105 were 60.23%, 45.34%, and 20.43%, respectively, and those of the validation populations ranged from 18.22% to 82.06%. For TN_s, the average values for parent JK, IR29, and KDML105 were 1.08, 1.00, and 1.00, respectively, and for TN_r, the average values were 1.00, 1.00, and 0.76, respectively. For the validation populations, TN_s and TN_r ranged from 0.75 to 3.00 and from 0 to 2.33, respectively.

**Table 3:** Phenotypic values of the three traits considering QTL1 in the two validation populations.

Population	Chr.	Trait	P1	P2	Progeny					
			Mean		Minimum	Maximum	Mean	SD	Kurtosis	Skewness
F_4_ (JK × IR29)	1	CMS	60.23	45.34	19.85	75.46	50.14	10.05	0.74	−0.25
	1	TN_s	1.08	1.00	1.00	1.91	1.18	0.21	1.74	1.39
	1	TN_r	1.00	1.00	0.75	1.75	1.06	0.18	4.34	1.84
F_3_ (JK × KDML105)	1	CMS	60.23	20.43	18.22	78.57	60.59	10.23	2.20	−1.05
	1	TN_s	1.08	1.00	0.75	2.33	1.20	0.30	1.79	1.49
	1	TN_r	1.00	0.76	0.00	2.33	1.06	0.30	3.22	0.71

P1 refers to JK, and P2 refers to IR29 or KDML105. CMS, cell membrane stability; TN, tiller number; s, stress phase; r, recovery phase.

**Table 4:** Phenotypic values of the three traits considering QTL2 in the two validation populations.

Population	Chr.	Trait	P1	P2	Progeny					
			Mean		Minimum	Maximum	Mean	SD	Kurtosis	Skewness
F_4_ (JK × IR29)	3	CMS	60.23	45.34	28.02	69.56	49.24	8.16	−0.12	−0.05
	3	TN_s	1.08	1.00	0.80	2.67	1.18	0.27	12.60	2.97
	3	TN_r	1.00	1.00	0.79	2.00	1.05	0.16	13.60	3.09
F_3_ (JK × KDML105)	3	CMS	60.23	20.43	19.52	82.06	60.36	10.42	2.04	−0.75
	3	TN_s	1.08	1.00	1.00	3.00	1.24	0.38	6.10	2.28
	3	TN_r	1.00	0.76	0.33	2.33	1.06	0.27	3.71	1.08

P1 refers to JK, and P2 refers to IR29 or KDML105. CMS, cell membrane stability; TN, tiller number; s, stress phase; r, recovery phase.

The trait correlation coefficients of the three phenotypes are shown in **Supplementary Figure 2** and **Supplementary Table 7**. In both validation populations, TN_r was substantially positively correlated with TN_s in the context of both QTL1 and QTL2. Similarly, in the F_3_ progeny, CMS also showed a strong positive correlation with TN_r in the context of both QTL1 and QTL2. Zhang et al. (2023) reported that TN shows a negative correlation with leaf electrolyte leakage (EL), indicating cell membrane damage, and is inversely related to CMS.

### 3.4 Validation of identified QTLs by genotyping segregating populations

**Figure 8:**
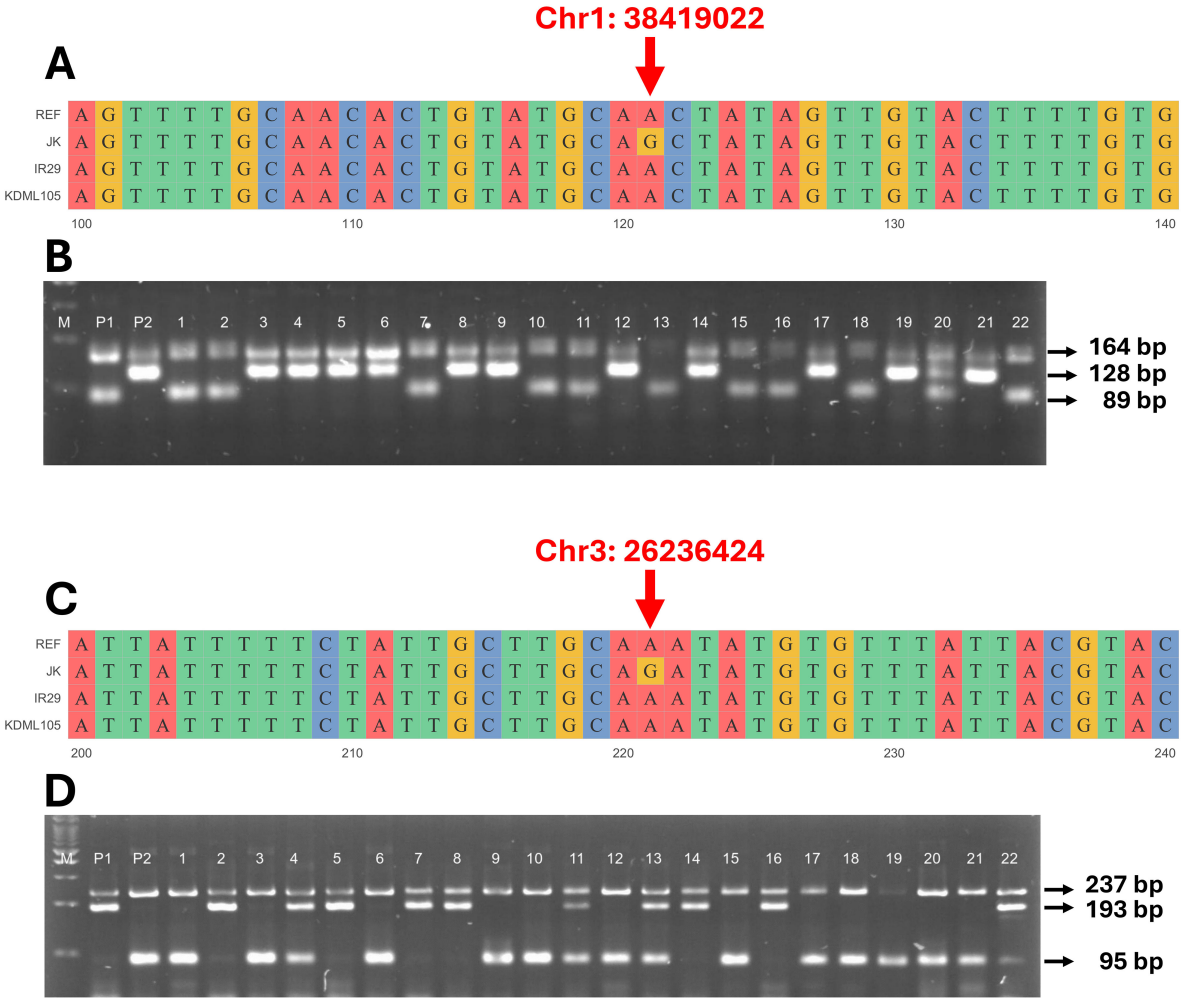
Genotyping of validation populations using allele-specific primers. Sequence alignment of target single-nucleotide polymorphisms (SNPs) on the QTL1 **(A)** and QTL2 **(C)** of JK, IR29, and KDML105 compared with the reference genome “Nipponbare”. Gel electrophoresis of QTL1 **(B)** outer primers (164 bp), JK (P1) allele (89 bp), and IR29 or KDML105 (P2) allele (128 bp). Lanes 1–22 are the individual progeny, and lane 20 is the heterozygous allele (three bands in lane). Gel electrophoresis of QTL2 **(D)** outer primers (237 bp), JK (P1) allele (193 bp), and IR29 or KDML105 (P2) allele (95 bp). Lanes 1–22 show the individual progeny, and lanes 4, 11, 13, and 22 show the heterozygous alleles (three bands in lane). M is a 100-bp marker ladder.

To confirm that the detected QTLs were related to the salt tolerance trait (CMS and TN_r), a molecular marker was generated based on the SNPs detected in these regions. As a result, primers were built for QTL1-SNP at position 38,419,022 bp on chromosome 1 (**Supplementary Figure 3**) and QTL2-SNP at position 26,236,424 bp on chromosome 3 (**Supplementary Figure 4**). The primers were developed to clearly distinguish the A and G alleles from two distinct genotypes (**Supplementary Table 8** and **Figures 8A, C**). The F_4_ and F_3_ progenies were evaluated to confirm QTL stability. PCR amplicons revealed differences in the size of each SNP; four different primers were used: (1) outer forward (OF), (2) outer reverse (OR), (3) inner forward (IF), and (4) inner reverse (IR). The OF and OR primers encompassed the entire range, confirming the existence of this segment. The OF-IR or IF-OR primer pairs provided unique products for each SNP. For QTL1, OF-OR generated 164 bp for the entire segment, OF-IR generated 98 bp for the P1 allele (JK), and IF-OR generated 128 bp for the P2 allele (IR29 or KDML105) (**Figure 8B**). For QTL2, OF-OR yielded 237 bp, OF-IR generated 193 bp for the P1 allele (JK), and IF-OR generated 95 bp for the P2 allele (IR29 or KDML105) (**Figure 8D**).

### 3.5 Phenotypic variance analysis

**Figure 9:**
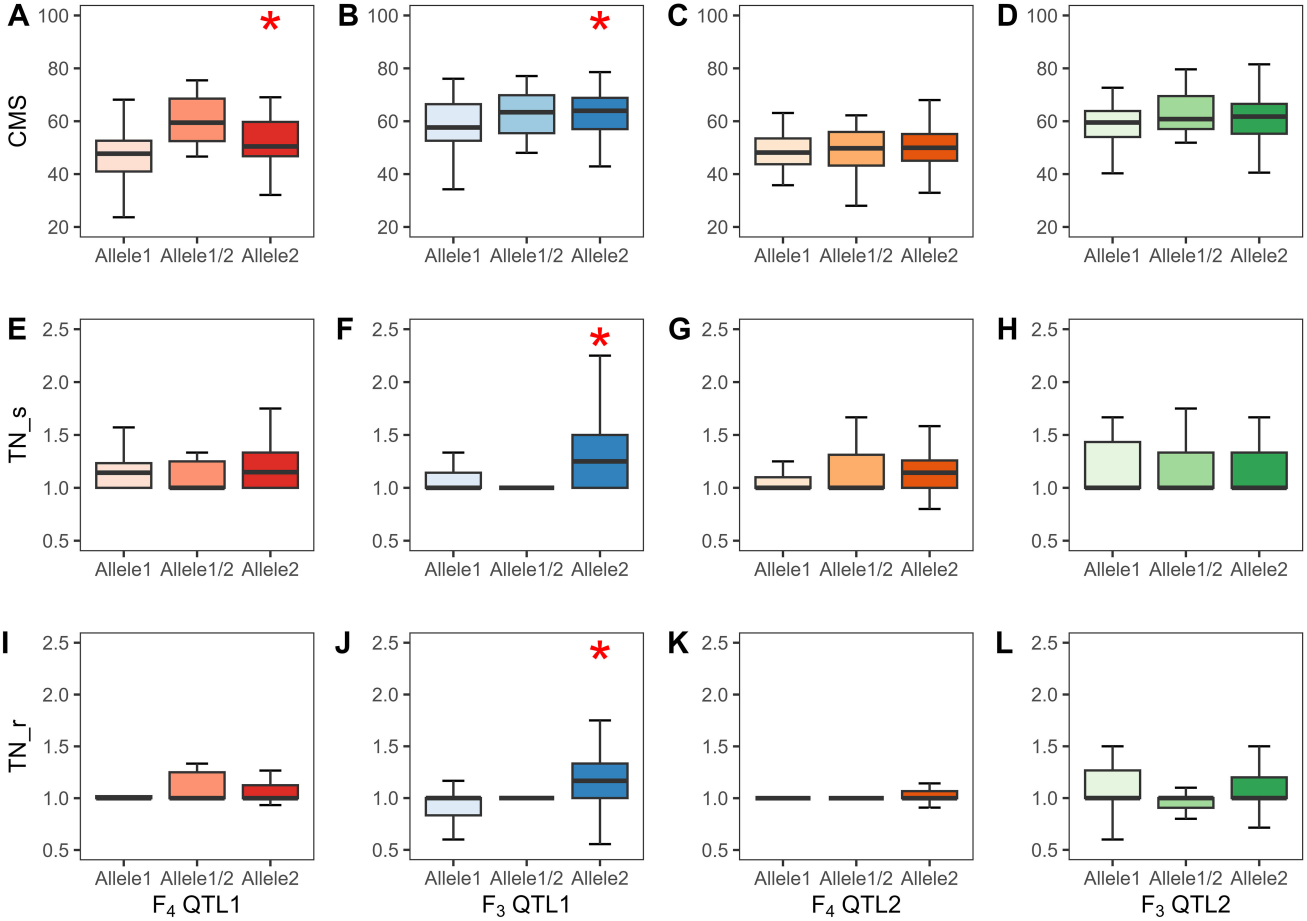
Phenotypes of QTL1 and QTL2 of the two validation populations (F_4_ and F_3_ populations). CMS, QTL1 **(A, B)** and QTL2 **(C, D)**; TN_s, QTL1 **(E, F)** and QTL2 **(G, H)**; TN_r, QTL1 **(I, J)** and QTL2 **(K, L)**. Allele1 refers to JK, allele1/2 implies a heterozygous allele, and allele2 indicates IR29 or KDML105 in the F_4_ and F_3_ populations, respectively. Significant QTLs at the 0.05 level are marked with *. The black line in the middle of the box shows the median. CMS, cell membrane stability; TN, tiller number; s, stress phase; r, recovery phase.

A total of 720 (considering QTL1) and 727 (considering QTL2) F_4_, and 824 (considering QTL1) and 808 (considering QTL2) F_3_ plants were phenotyped and genotyped, and the data were analyzed by the SSD method. The SSD method was used to maintain the genetic diversity of each line in the segregating progeny (Sleper and Poehlman, 2006); thus, the average phenotypic value of plants containing identical alleles in each line was calculated based on their similar genotype with at least three replicates. Subsequently, 82 and 84 lines based on the QTL1 and QTL2 genotyping of F_4_, respectively, and 153 and 159 lines based on the QTL1 and QTL2 genotyping of F_3_, respectively, were subjected to phenotypic variance analysis (**Supplementary Tables 2–5**). The phenotype and genotype correlations are represented as a box plot (**Figure 9**). Allele 1 with SNP G (alternative SNP) indicates the JK genotype, and allele 2 with SNP A indicates the IR29 or KDML105 genotype (similar to the reference genome “Nipponbare”). Linear regression was used to determine *R* ^2^ (shown as a percentage), which indicates the PVE by the QTL at a specific allele. For QTL1, CMS exhibited significant PVEs of 18.0% and 6.0% in F_4_ and F_3_, respectively. The PVEs of TN_s and TN_r were 16.2% and 20.4%, respectively, in the F_3_ population. The additive effect (AE) was negative, indicating that plants containing alleles from IR29 and KDML105 tolerate salt stress (**Table 5**; **Figure 9**; **Supplementary Table 9**).

**Table 5:** Phenotypic variance of identified *qCMS1-TN1* on chromosome 1 and *qCMS3* on chromosome 3 in the two validation populations.

QTL	Chr.	Trait	F_4_ (JK × IR29)			F_3_ (JK × KDML105)		
			AE	PVE (%)	*p*-value	AE	PVE (%)	*p*-value
QTL1 (*qCMS1-TN1*)	1	CMS	−3.25	**18.0**	0.0004	−2.70	**6.0**	0.0098
	1	TN_s	−0.01	0.5	0.8318	−0.12	**16.2**	0.0000
	1	TN_r	−0.02	2.1	0.4385	−0.14	**20.4**	0.0000
QTL2 (*qCMS3*)	3	CMS	−0.40	0.4	0.8590	0.75	1.3	0.3499
	3	TN_s	−0.05	4.5	0.1578	−0.03	1.5	0.3119
	3	TN_r	−0.01	0.7	0.7568	0.01	0.6	0.6152

AE, additive effect at a specific allele; positive values indicate effects of the JK allele; negative values indicate effects of IR29 or KDML105; PVE, percentage of total phenotypic variance explained by the QTL at a specific allele; bold PVE values represent significant QTLs at the 0.05 level; CMS, cell membrane stability; TN, tiller number; s, stress phase; r, recovery phase.

### 3.6 Comparison of validated versus previously reported QTLs

**Figure 10:**
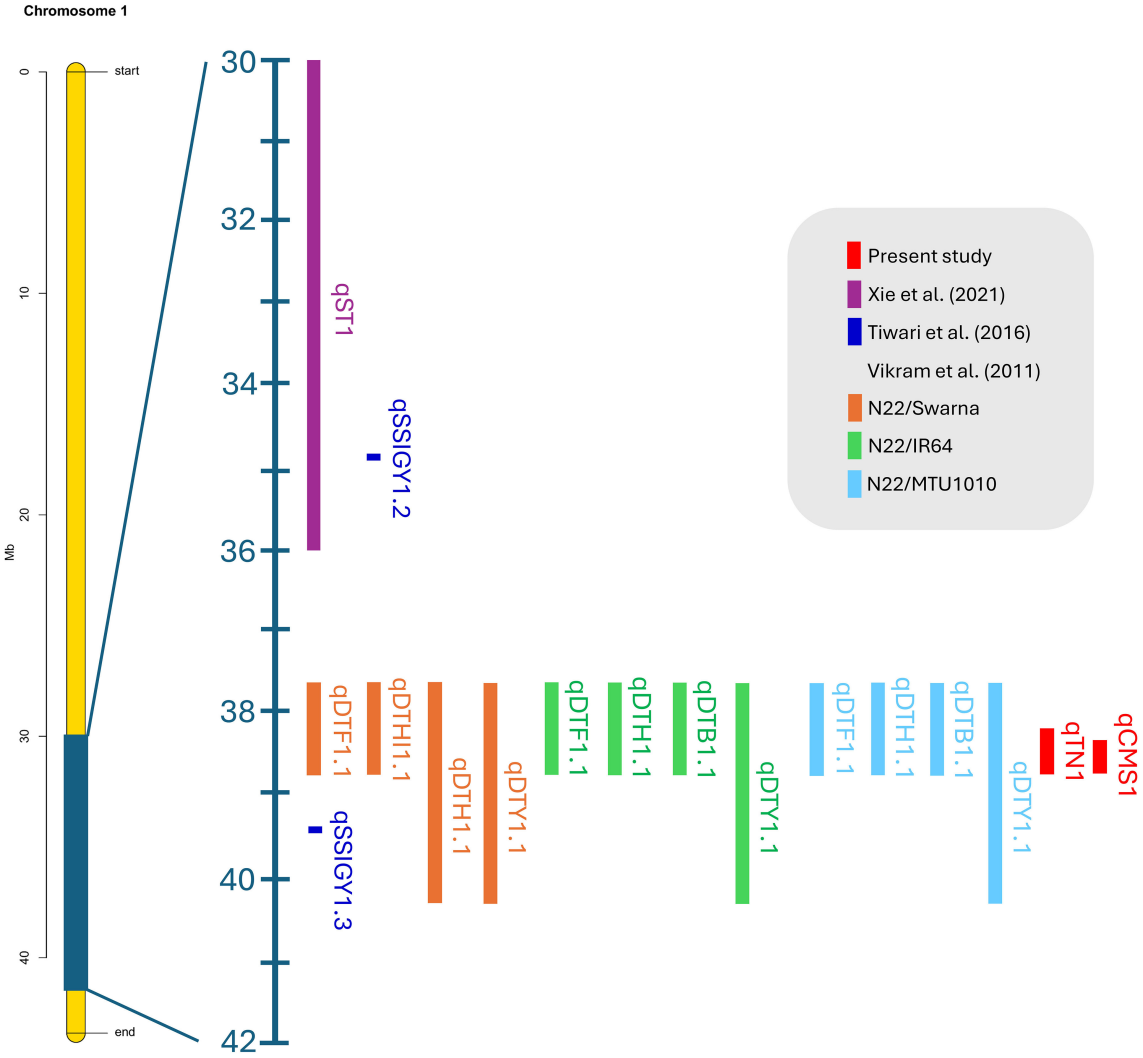
Colocation of verified rice quantitative trait loci associated with salt and drought stress on chromosome 1 was determined using the BSA-Seq technique.

Our validated QTLs were located at 38.26–38.95 Mb on chromosome 1 and 26.13–26.80 Mb on chromosome 3. Recent studies reported that the QTLs of various biparental crossings with different germplasms validated using BSA were colocalized in the overlapping region on chromosome 1. These QTLs, which correspond to salinity and drought stress, are associated with yield components, including the stress susceptibility index, seed germination rate, days to 50% flowering, plant height, biomass, and harvest index, which represent 3.5%–53.5% of the PVE (**Figure 10** and **Supplementary Table 10**).

## 4 Discussion

### 4.1 BSA-Seq for QTL mapping in an early segregating population

The MAS approach is an effective way to create new rice cultivars that resist salinity stress based on the introgression of salt-responsive genes/QTLs from donor to recipient lines (Miah et al., 2015; Rana et al., 2019). High-throughput genome sequencing can accelerate QTL mapping for identifying genomic loci related to traits of interest. The abundance of SNPs and InDels has been investigated at the genome level and can be applied to develop molecular markers (de la Fuente Cantó and Vigouroux, 2022). Here, we performed QTL-Seq mapping alongside the BSA technique based on the extreme opposite phenotypes of a biparental cross. The results of QTL-Seq mapping of the F_2_ generation revealed that two major salt-responsive QTLs were mapped by the CMS and TN traits on chromosomes 1 and 3, respectively, with approximately 500–1,000 SNPs present in the 1-Mb range of each region (**Tables 1**, **2**).

### 4.2 Phenotype distribution of two different genetic background progenies

To evaluate the stability of the identified QTLs, we established two validation populations with different genetic backgrounds, F_4_ (JK × IR29) and F_3_ (JK × KDML105), depending on JK, a salt-tolerant donor line. Some phenotypes exhibited a near-normal distribution. Skewness and kurtosis >1 imply a non-normal curve. If the skewness or kurtosis of the phenotype is between −1 and +1, the distribution is adequate (Mishra et al., 2019); however, QTL studies typically assume a normal distribution, although some traits may not follow this assumption (Li et al., 2015). Skewness indicated that the CMS of both progenies shifted to the right (negative value) (**Tables 3**, **4** and **Figures 6A, B**, **7A, B**), while the TN trait was skewed to the left (positive value) (**Figures 6C–F**, **7C–F**).

Compared with the F_4_ population, the F_3_ progeny tolerated salt better. This was demonstrated by the higher average CMS of approximately 60% in the F_3_ population compared with 50% in the F_4_ population. The mean CMS of F_3_ in QTL1 (60.59%) and QTL2 (60.36%) was similar to that of their parent JK (60.23%), whereas that of F_4_ was slightly lower than that of JK. Moreover, the mean TN of both progenies was slightly greater than that of JK (**Tables 3**, **4**). KDML105 is reportedly moderately sensitive to salt stress at the seedling stage, with a survival rate of 76.33% (Kanawapee et al., 2012). Therefore, crossing JK with KDML105 could result in the generation of plants that are highly tolerant to salt stress. Habila et al. (2021) discovered that the CMS of an F_2_ (JK × IR29) population grown in salted hydroponics had a bimodal distribution of polygenic traits, with a mean of 44% and positive correlation with TN. In the present study, the mean CMS of the F_2_ population was approximately 69.92% (**Supplementary Table 1**).

Furthermore, the AE considers the total effect of each allele that contributes to the phenotypic value of a particular trait. Consequently, the AE of each locus is expressed as the difference in mean values (Tan et al., 2022). In the present study, we considered the phenotype of plants that carry alleles from each parent (**Figure 8**): the donor line (JK) with a G allele, the recipient line (IR29 or KDML105) with an A allele, and the heterozygous allele with a G/A allele. The AE was calculated as [G/A-midpoint], with the midpoint determined as [G+A]/2. Thus, significant QTLs exhibited negative AE because G allele plants (JK allele) had phenotypic values lower than the midpoint. **Figure 9** reveals that plants that harbor JK allele at this position exhibit salt-sensitive phenotypes such as low CMS and TN values. The inverse AE of JK indicates that the allele that increases the desired phenotype (high CMS and TN) was from the recipient line (IR29 or KDML105)—AEs can be positive or negative values. Vikram et al. (2011) crossed drought-tolerant and high-yielding cultivars and detected QTLs located on the long arm of chromosome 1. In the same interval region as in the present study, these QTLs were found to have AEs with both positive and negative values.

### 4.3 QTL validation of the identified QTLs

QTL validation is frequently accomplished with two or more validation populations with distinct genetic architectures (Knoll and Ejeta, 2008; Singh and Singh, 2015). We validated the identified QTLs by phenotyping during both the stress (CMS and TN_s traits) and recovery (TN_r trait) stages.

Allele-specific primers were designed using an SNP that can distinctly differentiate the salt-tolerance allele (JK) from the salt-sensitive alleles (IR29 and KDML105). Kim et al. (2016) reported that to improve band separation during gel electrophoresis when the gap is small (<20 bp), the PCR product size of the ASP should be less than 120 bp. In the present study, we used at least 250 bp covering the target SNP with the gap between the products at approximately 30 bp, to design the ASP. The limitation was that if an indel was in this SNP region, the forward and reverse primers could not be constructed. With these criteria, two SNP markers were evaluated. The significant SNPs for the QTL1 and QTL2 markers at 38,419,022 bp on chromosome 1 and 26,236,424 bp on chromosome 3 were located in the intron regions of *LOC_Os01g66140* and *LOC_Os03g46390*, respectively, which were predicted by the Variant Effect Predictor (Arikit et al., 2019) (**Supplementary Figures 3** and **4**).

Chutimanukul et al. (2021) noted that in KDML105, chromosome segment substitution lines (CSSLs) containing different regions of DH212 between markers RM1003 and RM3362 show differential salt tolerance at the booting stage. This region overlaps with the QTL1 region identified in this study, which contains 71 genes, with 15 genes containing genic SNPs. Among these genes, *LOC_Os01g66890* (*OsBTBZ1*) was identified as a crucial gene responsible for the salt tolerance of CSSL16, functioning via ABA-dependent pathways (Saputro et al., 2023).

QTL1 (*qCMS1-TN1)* was stably expressed for 6%–18% of the PVE based on the CMS trait in the F_3_ and F_4_ populations but exclusively expressed for 16%–20% of the PVE based on the TN trait in the F_3_ population (**Table 5**). Correspondingly, TN_r was highly positively correlated with TN_s and CMS in the F_3_ population (**Supplementary Figure 2**).

The putative QTL on chromosome 3 with a minor phenotypic variance of less than 5% was not significantly different (**Table 5**). This could mean that the CMS and TN traits were not associated with this putative QTL. This finding is consistent with our QTL mapping, which revealed that the BSA mapping of the TN trait was only connected to QTLs on chromosome 1. The colocalization of *qCMS1* and *qTN1* on chromosome 1 was confirmed.

*qCMS-TN1* is a pleiotropic QTL in which a single locus contributes to multiple traits. In the present study, we found that the QTL1 locus likely controls the CMS, TN_s, and TN_r traits. A major QTL has a PVE of 25%–50% with a major effect; however, a minor QTL has a PVE of less than 25% with a slight and cumulative effect, which is the most common agronomic and physiological feature governed by numerous genes (Mackill and Ni, 2001; Singh and Singh, 2015). Because the PVE values of the significant QTLs were less than 25%, the validated QTLs were grouped with minor QTLs.

### 4.4 QTLs for cell membrane stability and tiller number

CMS, reflected by membrane injury, has been widely used to index plants under abiotic stress. Under salt stress, the cellular lipid membrane is destroyed by oxidative stress (Gao et al., 2023). QTLs related to CMS have been reported on chromosomes 1, 3, 7, 8, 9, 11, and 12 in rice under drought conditions and explained 13.4%–42.1% of the phenotypic variance (Tripathy et al., 2000). Hossain et al. (2015) reported that QTLs associated with TN at the flowering stage account for 11.3%–16.3% of the PVE on chromosomes 7 and 8 in F_2_ progeny and that TN positively correlates with panicle number (Deng et al., 2022) and length, grain yield, potassium content, and biomass. TN in rice directly affects grain yield, and the QTLs differ at each stage, from the seedling to the heading stages (Yan et al., 1998; Liu et al., 2010, 2012). Barnaby et al. (2022) detected QTLs for TN on the short arms of chromosomes 1 and 3 at various locations, accounting for 19%–33% of the PVE in 6-week-old F_10_ recombinant inbred lines of rice progeny. Pundir et al. (2021) reported that QTLs related to the number of productive tillers on chromosome 2 explained 5% of the variation in an F_2_ population. Moreover, Liu et al. (2010) argued that the potential maximum for TN QTLs across chromosomes 1, 3, 4, 5, 6, 7, 9, and 12 accounted for 2.54%–8.54% of the PVE in doubled haploid lines. The QTLs between the restriction fragment length polymorphism markers RZ730 and RZ801 on chromosome 1 represented approximately 35% of the PVE of the multiple-QTL model and were detected at every stage of growth (Yan et al., 1998). Finally, Lekklar et al. (2019a) indicated that the strongest positive correlations were observed between the TN per plant, panicle number, and unfilled grains. Collectively, these reports are consistent with our findings that CMS and TN are quantitative traits with small and cumulative effects.

QTL analyses frequently overestimate the impact of a QTL because the same dataset is used to identify and calculate its effect (% explained variance). QTLs with minor effects are challenging to identify in small mapping populations of approximately 100 individuals (number of lines of segregating population) unless they exceed the detection threshold by chance. This occurs due to a statistical sampling effect that amplifies their actual impact (Fischer, 2020).

In MAS, the process of selecting individuals carrying target genes involves the utilization of linked markers rather than relying solely on their observable phenotype within a segregating population. Consequently, the population may undergo screening at any developmental phase and across diverse environments (Khush and Brar, 2001). The utilization of molecular markers has the potential to facilitate breeders in optimizing parent and progeny selection processes with increased precision and efficiency. It is evident that an effective breeding program would profit from the capacity to decrease the number of plants in the initial generation (Rana et al., 2019; Marè et al., 2023). The purpose of employing markers in the early stages of a breeding program is to effectively eliminate plants that do not possess the desired gene combinations, consequently optimizing the subsequent stages of the program with reduced labor and space requirements, hence economizing the process. The concept of gene/QTL pyramiding involves the transfer of multiple genes of a particular trait into a single genotype. As a result, the application of MAS can significantly reduce both the cost and time requirements of selecting precise phenotypes that exhibit desired traits.

## 5 Conclusions

QTL-BSA-Seq was performed to map salt-responsive genes/QTLs in an F_2_ population, and the results were validated with two different genetic background crosses derived from the JK variety, a salt-tolerant local Thai rice variety. Candidate QTLs were mapped on chromosomes 1 and 3 based on CMS and TN traits, with CMS-bulk overlapping in a region similar to that of TN-bulk on chromosome 1. QTL validation confirmed the colocalization of QTLs for both traits on chromosome 1, which are pleiotropic QTLs in which one locus governs multiple traits. For future studies, using JK rice as a donor line for salt tolerance, we plan to utilize the identified QTL, *qCMS-TN1*, along with other markers for QTL pyramiding. This approach will be applied in MAS to improve rice salt tolerance, as well as to address drought stress.

## Data Availability

The original contributions presented in the study are publicly available. This data can be found here: https://www.ncbi.nlm.nih.gov/bioproject/PRJNA1148750.
