# Supplementary material for: QTL-Seq identified a genomic region on chromosome 1 for soil-salinity tolerance in F2 progeny of Thai salt-tolerant rice donor line “Jao Khao”
Source: Front Plant Sci. 2024 Aug 26;15:1424689. doi: 10.3389/fpls.2024.1424689 (PMC11385611; doi:10.3389/fpls.2024.1424689)
Supplement: Supplementary file 1 [file DataSheet1.docx]

Supplementary Material

QTL-Seq identified a genomic region on chromosome 1 for soil-salinity tolerance in F_2_ progeny of Thai salt-tolerant rice donor line ‘Jao Khao’

Prasit Khunsanit, Navarit Jitsamai, Nattana Thongsima, Supachitra Chadchawan, Monnat Pongpanich, Isabelle M. Henry, Luca Comai, Duangjai Suriya-Arunroj, Itsarapong Budjun, Teerapong Buaboocha^*^

*** Correspondence:** Teerapong Buaboocha: [teerapong.b@chula.ac.th](mailto:teerapong.b@chula.ac.th)

# Supplementary Figures and Tables

## Supplementary Figures

**
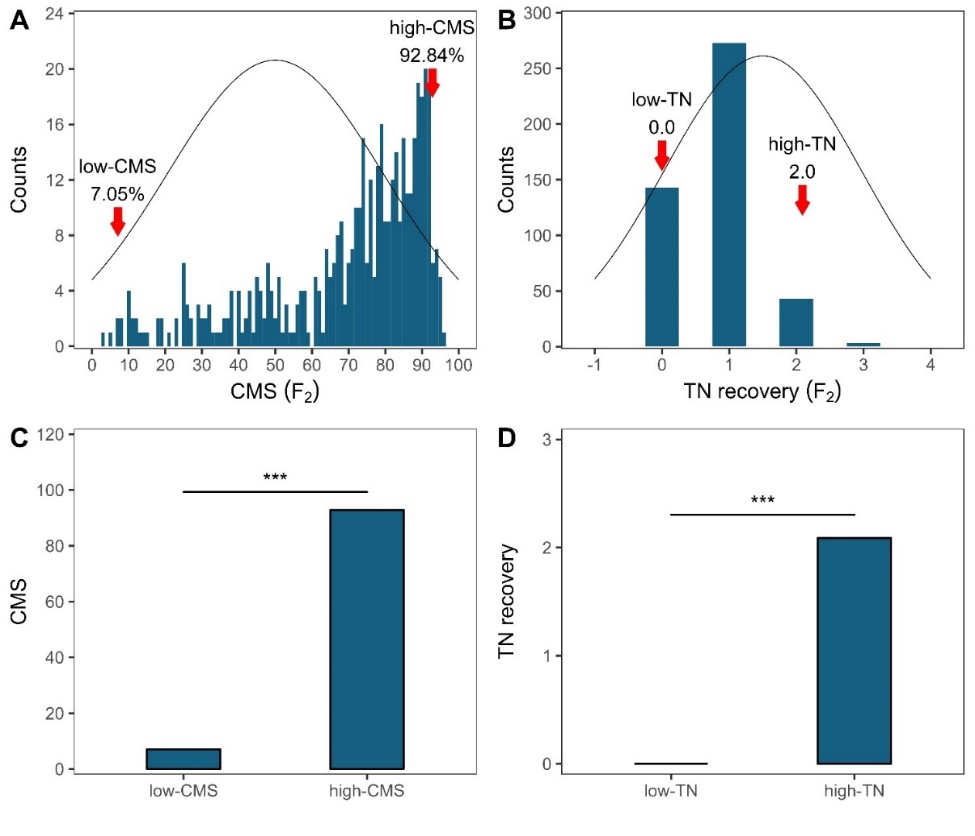
**

**Supplementary Figure 1.** Phenotypes of the F_2_ mapping population under salt stress conditions. Frequency distribution of cell membrane stability (CMS) (**A**) and tiller number (TN) (**B**) traits. The arrows indicate the means for the low- and high-bulk of each pool. Average phenotypic values of CMS during stress (**C**) and TN at the recovery stage (**D**). The bulks were equally sampled: high and low CMS bulk, *n* = 21, and high and low TN bulk, *n* = 28.


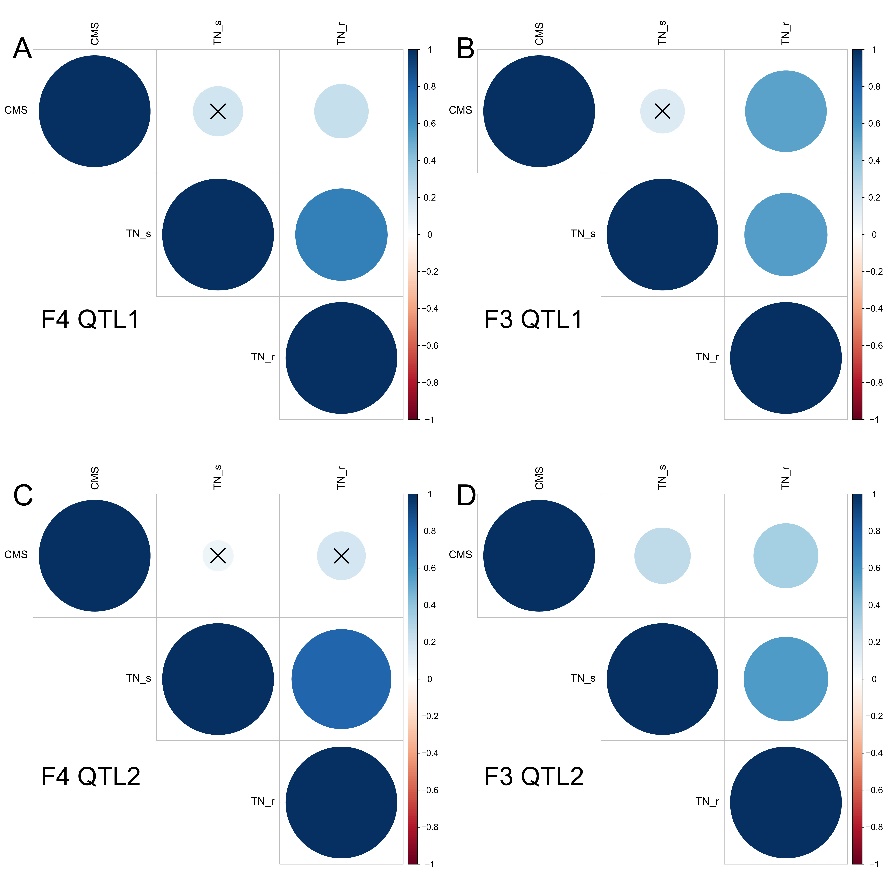


**Supplementary Figure 2.** Correlogram matrix of the three phenotypes. QTL1, F_4_ progeny (**A**) F_3_ progeny (**B**) and QTL2, F_4_ progeny (**C**) F_3_ progeny (**D**). Cells for which the correlation was not significant at *p* < 0.05 are marked ×. CMS, cell membrane stability; TN, tiller number; s, stress phase; r, recovery phase.


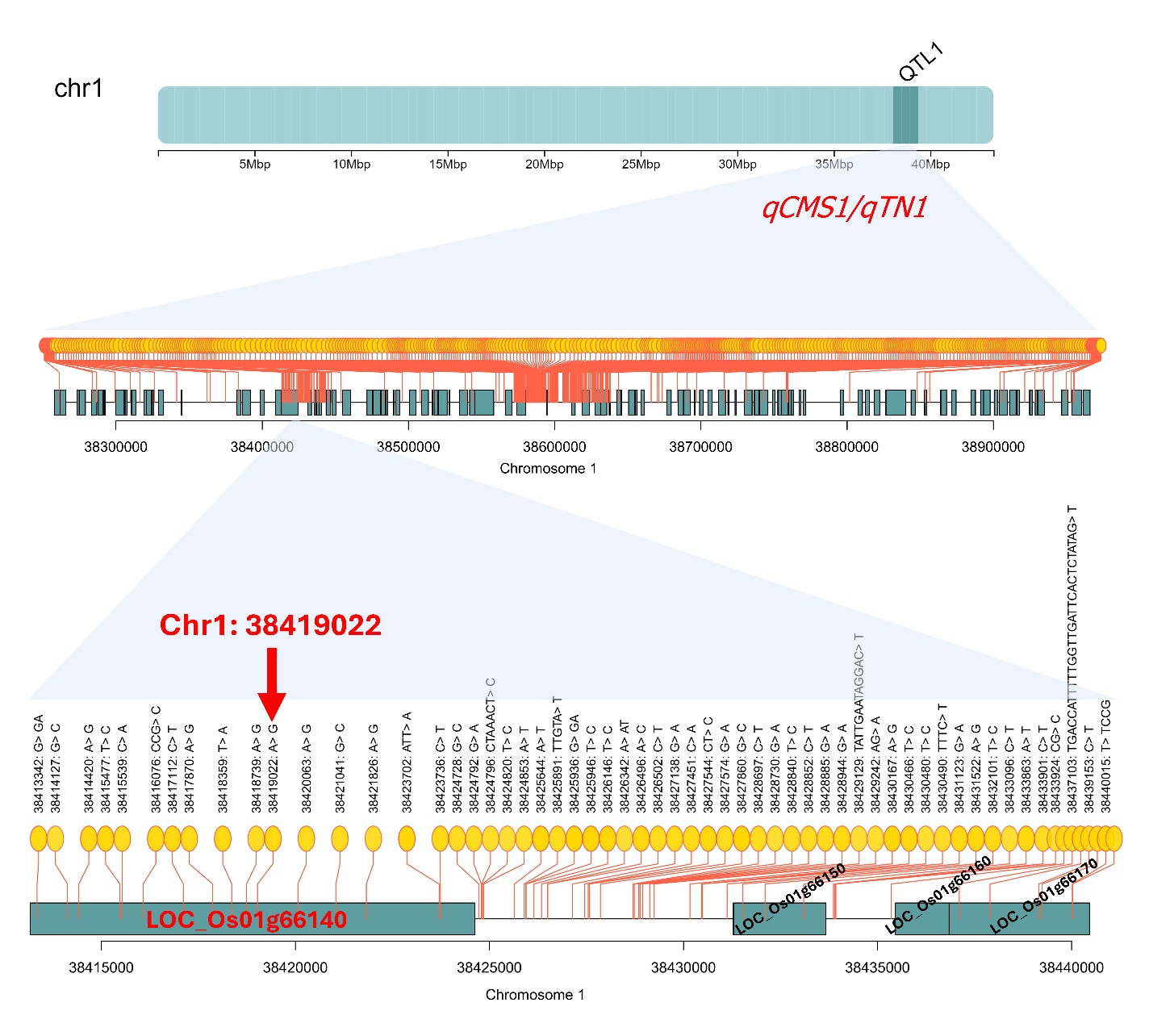


**Supplementary Figure 3.** Location of QTL1 (*qCMS1/qTN1*) on chromosome 1. The red lines represent significant single nucleotide polymorphism (SNP) locations, and the blue boxes indicate genes. The red arrow indicates the SNP position used for the marker.


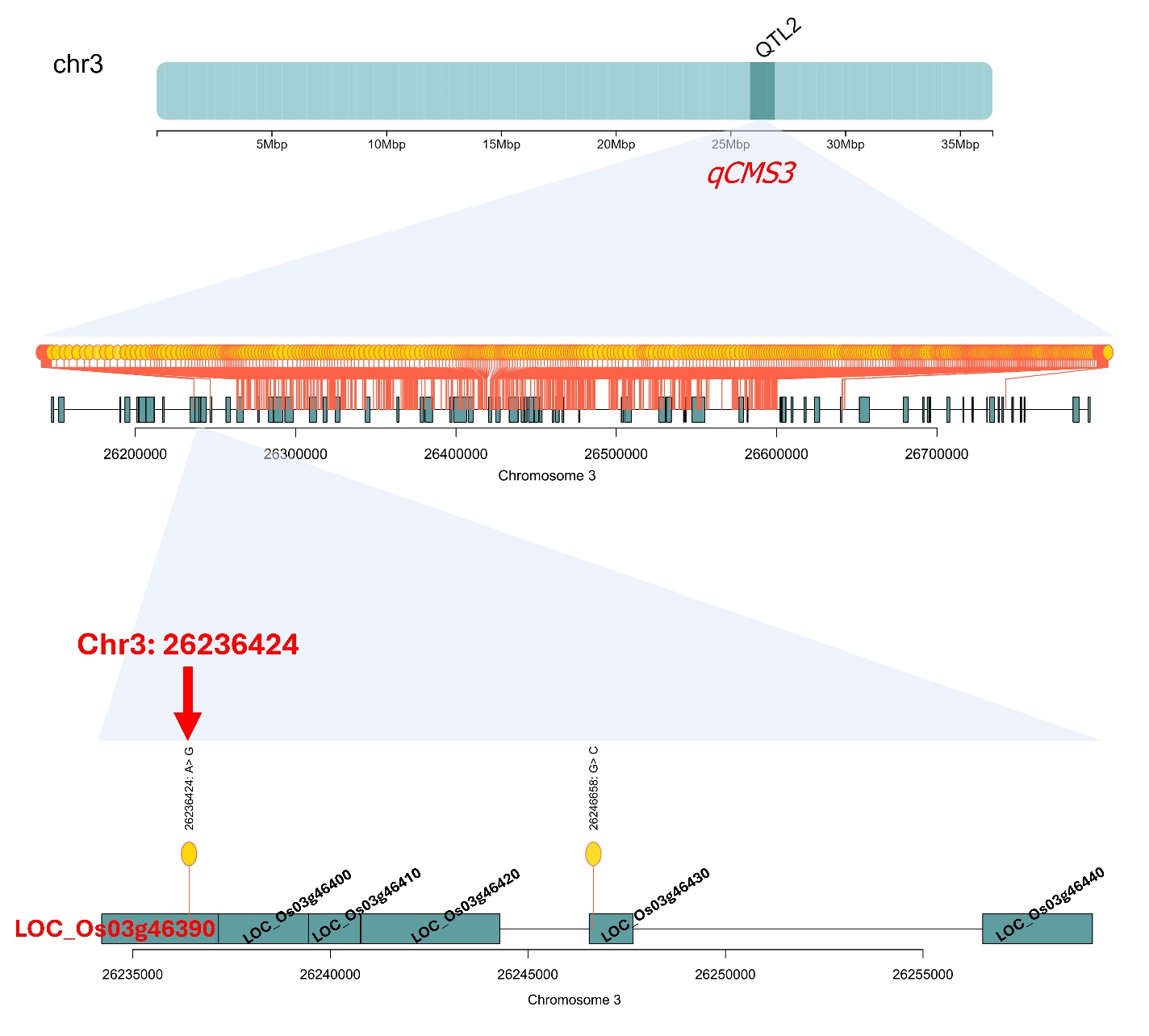


**Supplementary Figure 4.** Location of QTL2 (*qCMS3*) on chromosome 3. The red lines represent significant single nucleotide polymorphism (SNP) locations, and the blue boxes indicate genes. The red arrow indicates the SNP position used for the marker.

## Supplementary tables

**Supplementary Table 10.** Lists of rice QTLs verified to be associated with salt and drought stress by BSA-Seq.

|  | **Parent** | **Population**  **(size)** | **Marker type (number)** | **Growth stage** | **Screening condition** | **Pool size** | **QTL** | **trait** | **Chr.** | **Position, Mb** | **PVE, %** | **Reference** |
| --- | --- | --- | --- | --- | --- | --- | --- | --- | --- | --- | --- | --- |
| 1 | IR36/Weiguo | F_2_ (119) | KASP (25) | bud burst stage | 0.5% NaCl | 40 | qRSL7 | relative shoot length | 7 | 20.16-24.33 | 24.90 | (Lei et al. 2020) |
| 2 | Pokkali/KDML105 | RILs: F_7_ (160) | KASP (1) | seedling (14 days) | 150 mM NaCl | 33 | qST1.1 | salt injury score (SIS) | 1 | 5.0-5.50 | - | (Songtoasesakul et al. 2023) |
|  |  |  |  |  |  |  | qST10 |  | 10 | 17.75-18.25 | - |  |
|  |  |  |  |  |  |  | qST11 |  | 11 | 5.75-6.25 | - |  |
| 3 | CSR11/MI48 | RILs: F_10_, F_11_, F_12_ (216) | 50K SNP Chip | seedling (30 days) | moderate sodicity (pH ~ 9.5) and high sodicity (pH ~ 9.9) | 30 | qSSIGY1.1 | stress susceptibility index (SSI) for grain yield | 1 | 32.32 | - | (Tiwari et al. 2016) |
|  |  |  |  |  |  |  | qSSIGY1.2 |  | 1 | 34.97 | - |  |
|  |  |  |  |  |  |  | qSSIGY1.3 |  | 1 | 39.45 | - |  |
| 4 | Teng-Xi144/Long-Dao19 | F_2:3_ (1002) | 10K rice genotyping | three-leaf stage | 120 mmol/L NaCl | 50 | qSDS4-1 | survival days of seedlings (SDSs) | 4 | 24.62- 24.67 | 33.14 | (Lei et al. 2023) |
| 5 | Nipponbare/Haidao86 | F_2_ (138) | SNP/InDel (138) | seedling (14 days) | 2% NaCl, 32.9 dS/m | 20 | qST1 | seed germination rate | 1 | 30-36 | 9.27 | (Xie et al. 2021) |
| 6 | Jileng1/Milyang23 | RILs: F_10_ (253) | SNP (bin marker) | three-leaf stage | 10 dS/m | 20 | qW4SES6.1 | standard evaluating score (SES) | 6 | 6.65-7.63 | 15.8 | (Geng et al. 2023) |
|  |  |  |  |  |  |  | qW2SES11.1 |  | 11 | 4.13-4.84 | 17.4 |  |
| 7 | Sea Rice 86/Dianjingyou1 | F_2_ (186) | SSR (36) | trifoliate stage | 1.2% NaCl (w/v) | 30 | qST1.1 | relative salt damage rate | 1 | 11.24-12.26 | 62.6 | (Wu et al. 2020) |
| 8 | SR86/Nipponbare (Nip) SR86/9311 | F_2_ (600)  F_2_ (616) | - | three-leaf stage and heading stage | 150 mM NaCl | 30 | - | Surviving plants | 4 | 0.42-5.66 | - | (Gao et al. 2023) |
| 9 | Dongnong425/ Changbai10 | RILs: F_8_, F_9_ (180) | SSR/InDel | reproductive stage | 8.5 mM Na_2_CO_3_ (pH =9.0) | 20 | qATGW2-1 | 1000-grain weight | 2 | 21.47-22.01 | 5.15 | (Sun et al. 2021) |
|  |  |  |  |  |  |  | qATGW2-2 |  | 2 | 23.80-24.27 | 33.39 |  |
| 10 | N22/Swarna | F_3_ (292) | SSR (140) | reproductive stage | drained water from soil (drought stress) | 10% | qDTF1.1 | days to 50% flowering (DTF) | 1 | 37.84-38.88 | 3.5 | (Vikram et al. 2011) |
|  |  |  |  |  |  |  | qDTH1.1 | plant height (PH) | 1 | 37.84-40.24 | 32.6 |  |
|  |  |  |  |  |  |  | qDTY1.1 | grain yield (GY) | 1 | 37.84-40.24 | 13.4 |  |
|  |  |  |  |  |  |  | qDTHI1.1 | harvest Index (HI) |  | 37.84-38.88 | 6.2 |  |
| 11 | N22/IR64 | F_3_ (289) | SSR (140) | reproductive stage | drained water from soil (drought stress) | 10% | qDTF1.1 | days to 50% flowering (DTF) | 1 | 37.84-38.88 | 7.6 | (Vikram et al. 2011) |
|  |  |  |  |  |  |  | qDTH1.1 | plant height (PH) | 1 | 37.84-38.88 | 51.8 |  |
|  |  |  |  |  |  |  | qDTB1.1 | biomass (BIO) | 1 | 37.84-38.88 | 22.6 |  |
|  |  |  |  |  |  |  | qDTY1.1 | grain yield (GY) | 1 | 37.84-40.24 | 16.9 |  |
| 12 | N22/MTU1010 | F_3_ (362) | SSR (125) | reproductive stage | drained water from soil (drought stress) | 10% | qDTF1.1 | days to 50% flowering (DTF) | 1 | 37.84-38.88 | 5.5 | (Vikram et al. 2011) |
|  |  |  |  |  |  |  | qDTH1.1 | plant height (PH) | 1 | 37.84-38.88 | 53.5 |  |
|  |  |  |  |  |  |  | qDTB1.1 | biomass (BIO) | 1 | 37.84-38.88 | 30.3 |  |
|  |  |  |  |  |  |  | qDTY1.1 | harvest Index (HI) | 1 | 37.84-40.24 | 12.6 |  |

Underlined QTL represents co-location with the current study; F_2_, second filial generation; F_i_, *i*^th^ filial generation; F_2:3_, F_2_-derived F_3_; RILs, recombinant inbred lines; SSR, simple sequence repeat; KASP, Kompetitive allele-specific PCR.
